# P-1098. Implementation of a Patient-Driven Hand Hygiene Auditing Program in a Multi-Center Academic Healthcare System

**DOI:** 10.1093/ofid/ofaf695.1293

**Published:** 2026-01-11

**Authors:** Kevin Gibas

**Affiliations:** Brown University Health, Providence, RI

## Abstract

**Background:**

Proper hand hygiene is critical for minimizing the spread of infections within healthcare settings. Although extensive research has demonstrated the importance of high healthcare worker (HCW) hand hygiene compliance, many healthcare institutions face challenges in achieving optimal hand hygiene adherence. Low compliance may result in increased rates of healthcare-associated infections, prolonged hospitalizations, antimicrobial resistance, and higher healthcare costs.

**Figure:**
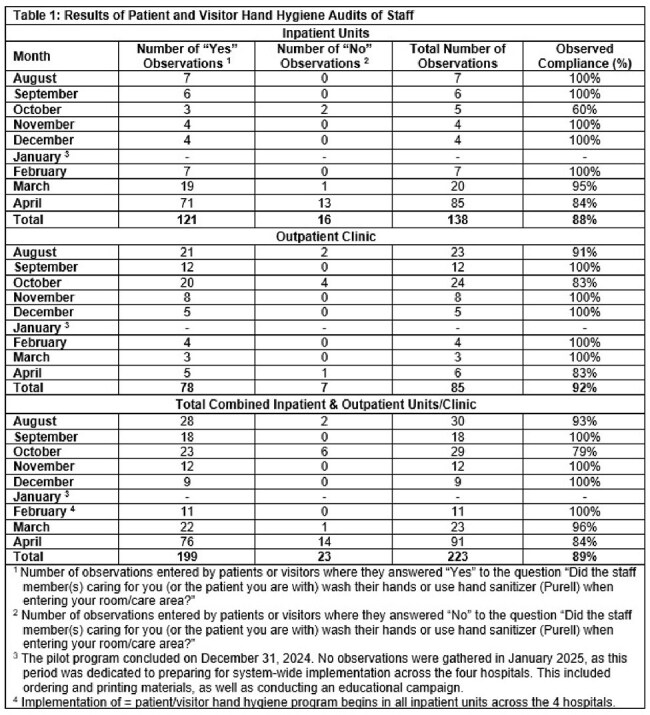

**Figure:**
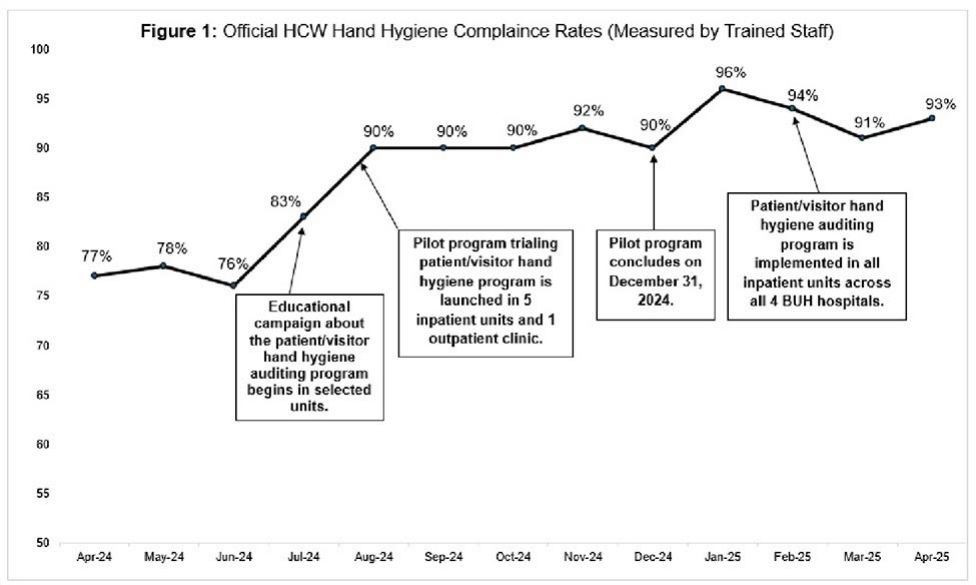

**Methods:**

We launched a novel program across 4 hospitals enabling patients/visitors to conduct audits of HCW hand hygiene compliance utilizing an online platform. An initial pilot was conducted from August to December 2024 in 1 outpatient clinic and 5 inpatient units. Following the pilot's completion, the program was expanded to all inpatient units across the 4 hospitals. Posters containing program information and a QR code linking to the audit form, were displayed in patient rooms and distributed to patients/visitors. The audit form contained 3 questions regarding their location (inpatient or outpatient), whether HCWs practiced hand hygiene (yes or no), and their comfort level in requesting staff to perform hand hygiene (yes or no). The form was accessible in English, Spanish, and Portuguese. Responses were recorded and anonymized in the online platform.

**Results:**

Patients/visitors have recorded 223 hand hygiene audits of staff: 85 in the outpatient clinic and 138 in the inpatient units (Table 1). HCW compliance observed by patients/visitors was 92% in the outpatient clinic and 88% in inpatient units (overall compliance of 89%). Figure 1 shows HCW hand hygiene compliance rates measured by trained BUH staff across the 4 hospitals from April 2024 through April 2025 in relation to the implementation of this program. After the start of education and implementation of this program, compliance generally increased and remained above pre-intervention levels. Approximately 76% of patients reported feeling comfortable requesting staff to perform hand hygiene.

**Conclusion:**

Preliminary data from this program demonstrates that engaging patients/visitors in hand hygiene interventions is practical and effective and warrants further use to improve HCW compliance and reduce healthcare-associated infections.

**Disclosures:**

All Authors: No reported disclosures

